# Multimodal MRI-Based Whole-Brain Assessment in Patients In Anoxoischemic Coma by Using 3D Convolutional Neural Networks

**DOI:** 10.1007/s12028-022-01525-z

**Published:** 2022-07-25

**Authors:** Giulia Maria Mattia, Benjamine Sarton, Edouard Villain, Helene Vinour, Fabrice Ferre, William Buffieres, Marie-Veronique Le Lann, Xavier Franceries, Patrice Peran, Stein Silva

**Affiliations:** 1 Toulouse NeuroImaging Center, Toulouse III Paul Sabatier University, Inserm, Toulouse, France; 2 Critical Care Unit, University Teaching Hospital of Purpan, Toulouse, France; 3 Laboratory of Analysis and Architecture of Systems, Toulouse III Paul Sabatier University, Centre National de Recherche Scientifique (CNRS), Institut National des Sciences Appliquees (INSA),, Toulouse, France; 4 Toulouse Cancer Research Center, Toulouse III Paul Sabatier University, Inserm, CNRS, Toulouse, France

**Keywords:** Coma, Cardiac arrest, Multimodal MRI, Deep learning, Convolutional neural networks

## Abstract

**Background:**

There is an unfulfilled need to find the best way to automatically capture, analyze, organize, and merge structural and functional brain magnetic resonance imaging (MRI) data to ultimately extract relevant signals that can assist the medical decision process at the bedside of patients in postanoxic coma. We aimed to develop and validate a deep learning model to leverage multimodal 3D MRI whole-brain times series for an early evaluation of brain damages related to anoxoischemic coma.

**Methods:**

This proof-of-concept, prospective, cohort study was undertaken at the intensive care unit affiliated with the University Hospital (Toulouse, France), between March 2018 and May 2020. All patients were scanned in coma state at least 2 days (4 ± 2 days) after cardiac arrest. Over the same period, age-matched healthy volunteers were recruited and included. Brain MRI quantification encompassed both “functional data” from regions of interest (precuneus and posterior cingulate cortex) with whole-brain functional connectivity analysis and “structural data” (gray matter volume, T1-weighted, fractional anisotropy, and mean diffusivity). A specifically designed 3D convolutional neuronal network (CNN) was created to allow conscious state discrimination (coma vs. controls) by using raw MRI indices as the input. A voxel-wise visualization method based on the study of convolutional filters was applied to support CNN outcome. The Ethics Committee of the University Teaching Hospital of Toulouse, France (2018-A31) approved the study and informed consent was obtained from all participants.

**Results:**

The final cohort consisted of 29 patients in postanoxic coma and 34 healthy volunteers. Coma patients were successfully discerned from controls by using 3D CNN in combination with different MR indices. The best accuracy was achieved by functional MRI data, in particular with resting-state functional MRI of the posterior cingulate cortex, with an accuracy of 0.96 (range 0.94–0.98) on the test set from 10-time repeated tenfold cross-validation. Even more satisfactory performances were achieved through the majority voting strategy, which was able to compensate for mistakes from single MR indices. Visualization maps allowed us to identify the most relevant regions for each MRI index, notably regions previously described as possibly being involved in consciousness emergence. Interestingly, a posteriori analysis of misclassified patients indicated that they may present some common functional MRI traits with controls, which suggests further favorable outcomes.

**Conclusions:**

A fully automated identification of clinically relevant signals from complex multimodal neuroimaging data is a major research topic that may bring a radical paradigm shift in the neuroprognostication of patients with severe brain injury. We report for the first time a successful discrimination between patients in postanoxic coma patients from people serving as controls by using 3D CNN whole-brain structural and functional MRI data.

*Clinical Trial Number* http://ClinicalTrials.gov (No. NCT03482115).

**Supplementary Information:**

The online version contains supplementary material available at 10.1007/s12028-022-01525-z.

## Introduction

Acute brain injury responsible for coma after cardiac arrest (CA) is a major cause of death and disability worldwide [1]. Despite the promise held by neuroscience research progress in the clinical treatment of patients with brain injury, the treatment of patients in coma has changed little over the last decade [2]. It has been suggested that the trouble to efficiently transfer neuroscience from bench to bedside in this field is mainly related to an inaccurate description of critical connectomes damages induced by CA [3]. Therefore, a timely, fine-grained characterization of coma-related functional and structural brain anomalies appears to be an unavoidable step to increase our understanding about the neural correlates of consciousness and pave the way toward the implementation of promising precision medicine approaches.

A growing body of literature supports the idea that brain multimodal magnetic resonance imaging (MRI), encompassing both structural MRI (sMRI) and functional MRI (fMRI) data, has the potential to fill this knowledge gap. Indeed, sMRI has demonstrated the usefulness of white matter fractional anisotropy (FA) [4] and gray matter morphometry [5] to predict neurological outcome after CA. In addition, fMRI studies have identified putative signatures of consciousness by using either static or dynamic resting-state connectivity [6–9]. Thereby, recent fMRI studies in patients in anoxoischemic coma have shed light on the role of frontal (mesial prefrontal cortex [mPFC]) and posterior parietal (precuneus [PreCun] and posterior cingulate cortex [PCC]) cortices as critical hubs within a putative brain mesocircuit underpinning consciousness emergence and maintain [10]. However, the complexity of multimodal MRI limits the timely interpretation and implementation in the clinic. Indeed, the vast majority of the reported MRI studies in patients in coma have explored only a reduced data set, either by using sMRI or fMRI techniques in isolation or by exclusively focusing on the assessment of hypothesis-driven brain regions of interest [4–9, 11].

There is an expanding interest in artificial intelligence–assisted neuroimaging interpretation to overcome the limits of subjective visual interpretation and to identify weak signals from complex multimodal neuroimaging data sets. In particular, convolutional neural networks (CNNs)—a deep learning network inspired by the animal visual system [12], in which connections between layers are made by sliding filters across the input data—have demonstrated to be highly efficient for analyzing composite images. CNNs can provide low-level to high-level representations of data and can perform automatic and task-optimized “feature extraction” followed by classification, whereby the algorithm itself (and not the programmer) defines which features of the signal are relevant for an accurate classification [13]. Nevertheless, understanding a CNN’s decision-making process is key to reliable and reproducible results [14]. Described as black boxes, CNNs are being intensively investigated by the scientific community to gain insight on their functioning. To this end, several techniques have been developed, from visualizations of layer activation to the creation of self-explaining models [15]. In the medical domain, CNNs have shown great performances for a range of tasks, such as age prediction and classification of neurodegenerative diseases, by using neuroimaging data [16–18]. However, to the extent of our knowledge and despite their potential, CNNs have never been used for weak signal detection from multimodal brain MRI in patients in coma.

In this present proof-of-concept study, we sought to develop and validate a CNN model to leverage complex multimodal 3D MRI whole-brain times series for an early evaluation of brain damages related to anoxoischemic coma. The CNN model’s performance will be analyzed, and to gain insight on CNN functioning, we will also explore the specific contribution of each MRI modality to the proposed 3D CNN architecture. Hence, to identify regions of the input most relevant for CNN prediction, visualizations of filter activation will be provided for each magnetic resonance (MR) index. Finally, we will investigate the potential added value for the patient’s neuroprognostication of CNN misclassifications.

## Methods

### Study Design

This prospective study was undertaken at the intensive care unit affiliated with the University Hospital (Toulouse, France), between March 2018 and May 2020. Patients were treated according to current guidelines by physicians blinded to neuroimaging data. To avoid confounding factors, all patient assessments were conducted at least 2 days (4 ± 2 days) after complete withdrawal of sedation and were performed in normothermia condition. Patients were included in the study after they had a behavioral assessment with Glasgow Coma Scale (GCS) [19] and had been diagnosed as been in coma (GCS score at the admission to hospital < or = 6 with motor responses < 6) induced by CA. A patient’s neurological outcome was assessed 3-months after the hospital admission (Coma Recovery Scale revised [CRS-R]) [20]. Over the same period, healthy volunteers, were recruited and included if they had normal neurological examination results and no history of neurological or psychiatric disorder. Our study was approved by the Ethics Committee of the University Teaching Hospital of Toulouse, France (2018-A31). Informed and written consent to participate to the study was obtained from the participants themselves in the case of healthy participants and from legal surrogate of the patients. Clinical trial identifier: NCT03482115.

### Population

Details of the recruitment and treatment have been described elsewhere [8]. Patients were included in the study after they had a behavioral assessment with GCS and had been diagnosed as being in coma (GCS score at the admission to hospital < or = 6 with motor responses < 6) as a consequence of a primary anoxoischemic brain injury. Exclusion criteria was a patient experiencing head motion of more than 3 mm in translation and 3° in rotation during MRI acquisition.

### Clinical Outcome

All patients were followed up until death or 3 months after CA. The principal outcome measure was the CRS-R [10], which was assessed by raters blinded to MRI data. Following current guidelines for the diagnosis of disorders of consciousness in patients with severe brain injury [1, 2], CRS-R allowed the diagnosis of minimally conscious state (MCS), which was defined according to the identification of command-following, intelligible verbalization, or intentional communication abilities, and vegetative state/unresponsive wakefulness syndrome (VS/UWS), corresponding to fully awake but unaware patients. Moreover, as previously reported [2], MCS was further classified as “ + ” or “ − ” according to the detection of a patient’s command-following. Eventually, among survivors, “MCS + ” or “MCS –” were defined as favorable outcome, whereas VS/UWS or death were defined as unfavorable outcome (Supplementary Table 1, Supplementary Fig. 1).

### MRI Data Acquisition

In all participants, 11 min of resting-state fMRI (rs-fMRI) data were acquired on the same 3 T MR scanner (Intera Achieva; Philips, Best, the Netherlands). Monitoring of vital measures was performed by a senior intensivist throughout the experiment (BS, HV, FF, WF, SS). High-resolution anatomical image, using 3D T1-weighted sequence and apparent diffusion coefficient were also acquired. Concerning sMRI data, gray matter morphometry was applied on 3D-T1-weighted images by using voxel-based morphometry [5], obtaining an estimation of gray matter volume (GM). To study white matter integrity, DTI models were created to fit at each voxel, generating FA and mean diffusivity (MD) maps [4]. Cortical frontal (mPFC) and posterior parietal (PreCun and PCC) regions of interest (ROI) were defined to be used in ROIs vs. whole-brain functional connectivity analysis [8]. rs-fMRI data were preprocessed using Statistical Parametric Mapping (version SPM 12; http://www.fil.ion.ucl.ac.uk/spm/). As described elsewhere [6–8], fMRI images were realigned, slice-time corrected, coregistered to each patient’s T1-weighted image and normalized to standard stereotaxic anatomical Montreal Neurological Institute space (Table 1).

**Table 1:** Architecture of the proposed 3D CNN

Layer	Filters	Filter size	Stride	Units	Following layers
Conv3D	32	(3, 3, 3)	(1, 1, 1)	–	BN + ELU
AveragePooling3D	–	(3, 3, 3)	(3, 3, 3)	–	–
Conv3D	64	(3, 3, 3)	(1, 1, 1)	–	BN + ELU
Conv3D	64	(3, 3, 3)	(1, 1, 1)	–	BN + ELU
AveragePooling3D	–	(2, 2, 2)	(2, 2, 2)	–	–
Conv3D	128	(3, 3, 3)	(1, 1, 1)	–	BN + ELU
Conv3D	128	(3, 3, 3)	(1, 1, 1)	–	BN + ELU
Conv3D	128	(3, 3, 3)	(1, 1, 1)	–	BN + ELU
AveragePooling3D	–	(2, 2, 2)	(2, 2, 2)	–	–

**Flatten**					
FCL	–	–	–	512	BN + ELU + Dropout (0.5)
FCL	–	–	–	512	BN + ELU + Dropout (0.25)
FCL	–	–	–	512	BN + ELU + Dropout (0.25)
FCL	–	–	–	512	BN + ELU
FCL	–	–	–	2	Softmax
Total parameters		4,432,386	Trainable parameters		4,427,202

Convolutional layers (Conv3D) are characterized by number of filters along with filter size and stride, followed by batch normalization (BN) and exponential linear unit (ELU) activation. Filter size and stride are detailed for pooling layers (AveragePooling3D). The number of units is provided for each fully connected layer (FCL). Dropout probabilities are specified for dropout layers. Prior to FCLs, the output from convolutional layers is transformed in a one-dimensional array (Flatten). CNN, convolutional neuronal network

### 3D CNN Implementation

Convolutional neural networks can process high-dimensional arrays while preserving their spatial relationship. Relying on local connections, CNNs exploit data structure to make learning more efficient [21]. A typical CNN is composed by a sequence of alternating layers, denoted as convolutional and pooling, followed by an artificial neural network responsible for classification [22]. Convolutional layers are named on the operation they perform, i.e., convolution, which allows them to scan the input by using a filter—a matrix of numbers—obtaining the so-called feature maps. Each filter retrieves specific characteristics in the image, e.g., edges or shapes. Indeed, convolutional filters are learned during training to optimize the task at hand. This frees the CNN from hand engineered feature extraction, achieving outstanding results. Pooling layers instead allows the CNN to aggregate the information from convolutional ones, enabling dimensionality reduction and invariance to distortions. The higher the number of layers, the deeper the CNNs and the more abstract their representations of data [13].

**Fig. 1:**
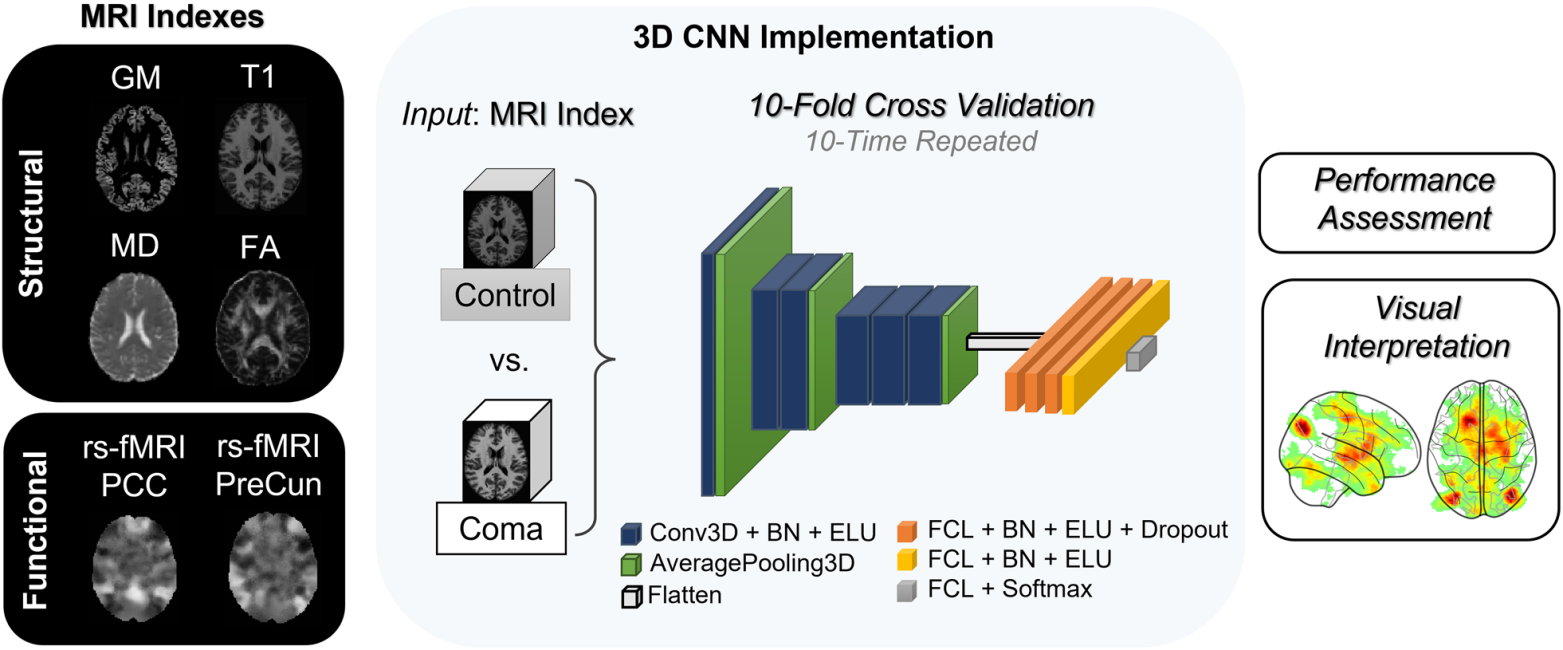
Methods overview. Structural and functional magnetic resonance (MR) indices from the set of controls (*n* = 34) and patients in coma (*n* = 29) were assessed to perform binary classification by using a 3D convolutional neuronal network (CNN) in a 10-time repeated tenfold cross-validation. The 3D CNN model is schematized with fundamental building blocks. Feeding as input each MR index, we examined their discriminant power by using standard evaluation metrics and visualization maps to discover the most relevant voxels taken into account for CNN prediction. AveragePooling3D, average pooling layer, BN, batch normalization, Conv3D, convolutional layer, Dropout, dropout layer, ELU, exponential linear unit activation, FCL, fully connected layer, Flatten, output from the convolutional part reshaped in a 1D array, Softmax, softmax activation

Once the feature extraction completed, classification was performed by a multilayer perceptron taking as input the learned features. Multilayer perceptrons are feedforward neural networks that map an input to an output via nonlinear functions. This mapping consists of finding the optimal weights (i.e., matrices of numbers) to match the output by optimizing a loss function. The latter defines the truthfulness of the outcome with respect to the predicted label, using error backpropagation for supervised tasks [23]. The processing units of multilayer perceptrons are the well-known artificial neurons, whose functioning is based on the biological neuron. In the same way as a biological neuron does, an artificial neuron acquires inputs, processes them, and then passes the processed outputs to neighboring neurons [24, 25]. An overview of the approach adopted in this study is represented in Fig. 1, underlining the different MR indices fed as input to the 3D CNN.

The architecture of the developed 3D CNN is detailed in Table 2. It was inspired by AlexNet and VGGNet revised in a 3D adaptation [26, 27]. In the proposed model, convolutional layers presented filter size of 3 and stride = 1 for all dimensions and increasing number of features maps through the network (32, 64 and 128), followed by batch normalization and exponential linear unit activation. Batch normalization favors the decrease of internal covariate shift, thus accelerating learning [28]. The valid method was preferred for convolutional layers instead of padding [29]. Average pooling with filter size of 2 was applied except for the first pooling layer with filter size of 3, considering stride equal to filter size for all dimensions. After transforming the convolutional output in a one-dimensional array, the fully connected part processes it. The first four fully connected layers (FCLs) were composed by 512 units with exponential linear unit activation. Among them, the first three FCLs were followed by dropout, a regularization technique able to limit overfitting by randomly dropping a certain percentage of units [30]. The last FCL was composed by two units to allow for binary classification with Softmax as activation function.

**Table 2:** Model classification performance

MR index	AUC (95% CI)	Accuracy	Sensitivity	Specificity	PPV	NPV
GM	0.84 (0.13, 0.81–0.86)	0.84 (0.13, 0.81–0.86)	0.72 (0.24, 0.67–0.76)	0.96 (0.10, 0.94–0.98)	0.95 (0.13, 0.92–0.98)	0.82 (0.15, 0.79–0.85)
T1	0.82 (0.15, 0.79–0.85)	0.82 (0.15, 0.79–0.85)	0.77 (0.25, 0.72–0.82)	0.87 (0.18, 0.83–0.91)	0.86 (0.19, 0.82–0.90)	0.84 (0.16, 0.81–0.87)
MD	0.89 (0.13, 0.86–0.91)	0.89 (0.13, 0.86–0.91)	0.82 (0.23, 0.78–0.87)	0.95 (0.13, 0.92–0.97)	0.95 (0.13, 0.92–0.97)	0.88 (0.15, 0.85–0.91)
FA	0.92 (0.11, 0.89–0.94)	0.92 (0.11, 0.89–0.94)	0.86 (0.20, 0.83–0.90)	0.97 (0.11, 0.95–0.99)	0.97 (0.10, 0.95–0.99)	0.91 (0.13, 0.88–0.94)
rs-fMRI PCC	*0.96 (0.08, 0.94–0.98)*	*0.96 (0.08, 0.95–0.98)*	*0.95 (0.12, 0.93–0.97)*	*0.97 (0.09, 0.95–0.99)*	*0.97 (0.08, 0.96–0.99)*	*0.96 (0.09, 0.95–0.98)*
rs-fMRI PreCun	0.90 (0.12, 0.88–0.93)	0.90 (0.12, 0.88–0.93)	0.88 (0.20, 0.84–0.91)	0.93 (0.14, 0.90–0.96)	0.93 (0.13, 0.91–0.96)	0.92 (0.13, 0.89–0.94)

Evaluation metrics obtained for each MR index (T1, FA, GM, MD, fMRI-PreCun, fMRI-PCC) used to train the 3D CNN obtained on the test set from 10-time repeated tenfold cross-validation. Results are provided as mean (SD, 95% CI). Best performances are highlighted in italic

CI, confidence interval, FA, fractional anisotropy, GM, gray matter volume, MD, mean diffusivity, MR, magnetic resonance, MRI, magnetic resonance imaging, NPV, negative predictive value, PCC, posterior cingulate cortex, PPV, positive predictive value, PreCun, precuneus, rs-fMRI, resting-state functional MRI, SD, standard deviation, T1, T1-weighted

The model trained for 100 epochs using mini-batch gradient descent with eight samples as batch size and applying L2 regularization of factor equal to 0.0005. Adam optimizer was selected with initial learning rate of 0.00005 but decreasing after five epochs without any progress in performance [31]. The employed loss function was categorical cross-entropy [25].

Keras [29] and Tensorflow [32] libraries (versions 2.2.4 and 1.13.1, respectively) were exploited for model implementation together with a graphical processing unit Nvidia Quadro RTX 6000.

Concerning input data, the set of controls and patients in coma available for each MR index was fed as raw input to the CNN to evaluate their individual informative content. Images were normalized according to training set values to lie in the range from 0 to 1. Given the limited sample size, tenfold cross-validation was performed as customary in the neuroimaging field [33], repeated 10 times to reduce performance bias.

### Visual Interpretation

To shed light on CNN classification, we applied a previously reported visualization technique [34]. According to this method, the output from each convolutional layer was retrieved. Only positive activation values were considered for the classification. Because data dimension is reduced going deeper in the network, these visualization maps were interpolated to match the input size. To merge results, first the maps were averaged over filter number for each convolution and then over the total number of convolutions to obtain the final visualization map.

Considering only the maps obtained from correctly classified samples of the training set, we computed the absolute difference between the normalized averaged maps per class to highlight the most significant voxels.

Under the assumption that low activation values contributed less to the prediction, we applied a threshold equal to half of the maximum value to each visualization map.

### Statistical Analysis

Continuous data are expressed as mean ± standard deviation and/or median (range), according to their distribution (Kolmogorov–Smirnov test). Categorical variables were expressed as numbers and percentages. Sensitivity, specificity, and diagnostic accuracy were calculated using standard formulas as well as positive predictive value and negative predictive value. Receiver operating characteristic curves were calculated for each predictive model, and the highest sum of sensitivity and specificity was considered as being the optimal threshold. All *p* values were two-tailed, and statistical significance was defined as a *p* value of less than 0.05.

Misclassified patients were examined in relation with the known outcome at 3 months after the primary brain injury event. Hence, we computed the false negative (FN) good outcome rate, i.e., the percentage of FN having a good outcome over the total FN count. Furthermore, we applied majority voting, considering all predictions regardless of the MR index: the final label associated with the sample was dictated by the most scored prediction [26, 27]. This allowed to check whether merging outcomes from different MRI modalities could enhance performance.

## Results

### Population

A total of 35 patients in anoxoischemic coma were prospectively identified at the time of hospital admission. Among them, five did not fulfill at least one inclusion and one withdrew consent. The final cohort consisted of 29 patients, aged 62.0 (51.6 to 75.0) years, of whom 15 (49.2%) were women. Patients have a GCS of 6 (3 to 7) before sedation onset. The mean intensive care unit (ICU) stay was 15 ± 10 days. Thirty-four age-matched healthy volunteers 61.0 (51.0 to 72.1) were also included in the study (Supplementary Table 1, Supplementary Fig. 1).

### Model Performance

Performances of the proposed 3D CNN were remarkable for all MR indices. However, some difference exists among the considered MR indices, of which the results are outlined in Table 2. Overall, structural indices presented lower accuracy and area under the curve (AUC) compared with those of functional indices. Indeed, the best performance was obtained by the functional indices, in particular by rs-fMRI PCC (accuracy of 0.96). In contrast, poorer performance was observed by the structural indices, with the lowest accuracy reached by T1 index (accuracy of 0.82).

In general, sensitivity was lower compared with specificity, regardless of the MR index. Positive predictive value was instead systematically higher than negative predicted value.

It is worth noting that performances on patients were comparable between GM and T1 but was rather low with respect to the other indices with sensitivity not higher than 0.77.

### Classification Errors

**Fig. 2:**
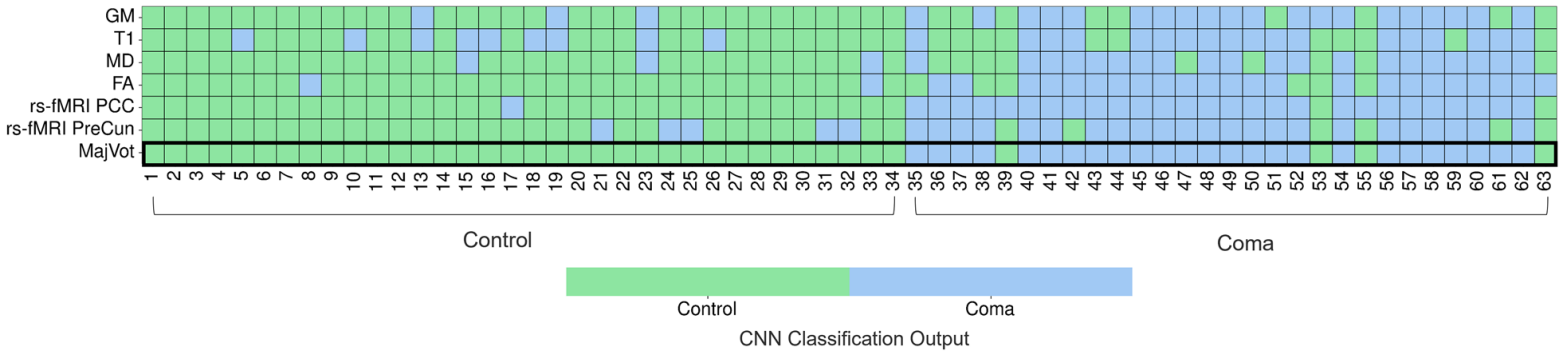
Individual classification according to magnetic resonance imaging (MRI) indices. Analysis of misclassified samples was conducted on the basis of model performance. Controls and patients in coma were associated with their classification label assigned by the 3D convolutional neuronal network (CNN) according to magnetic resonance (MR) index. Majority voting (MajVot) was computed to assess whether the individual MR index performance on each sample could be improved considering the most scored classification output among all MR indices. This was indeed the case for controls, all correctly classified with MajVot. Regarding patients in coma, MajVot was second only to rs-fMRI PCC, totaling only two misclassified patients instead of four. FA, fractional anisotropy, GM, gray matter volume, MD, mean diffusivity, PCC, posterior cingulate cortex, PreCun, precuneus, rs-fMRI, resting-state functional MRI, T1, T1-weighted

Examining misclassified samples in Fig. 2, majority voting allowed to correctly classify all controls. Overall, functional indices were better at classifying both patients and controls. Focusing on patients in coma, the majority voting index outperformed every individual MR index. Among the structural indices, T1 presented the highest false negative (FN) and false positive (FP) counts whereas FA presented the smallest number of misclassified samples. For further details, please see Tables 2 and 3.

**Table 3:** Relationship between coma patient misclassification and 3-month neurological outcome

MR index	FN good outcome rate (%)	Good outcome FN	Total FN
GM	44	4	9
T1	64	7	11
MD	56	5	9
FA	50	3	6
rs-fMRI PCC	*100*	*2*	*2*
rs-fMRI PreCun	67	4	6

MR indices are detailed with corresponding number of misclassified coma patients (FN). Each FN was associated with the known outcome after 3 months from the comatose event to find out whether there can be some relationship with patients having recovered from coma, thus classified as controls. Best results are highlighted in italic

FA, fractional anisotropy, FN, false negative, GM, gray matter volume, MD, mean diffusivity, MR, magnetic resonance, MRI, magnetic resonance imaging, PCC, posterior cingulate cortex, PreCun, precuneus, rs-fMRI, resting-state functional MRI, T1, T1-weighted

Considering a patient in postanoxic coma’s neuroprognostication, we investigated the potential relationship between patient outcome at 3 months after the CA and misclassified patients in coma (FN). In fact, we explored the hypothesis that patients who showed a favorable neurological outcome were those presenting similar structural and/or functional connectomes to healthy patient and were therefore prone to be classified as controls despite their clinical status. Indeed, about half of misclassified patients presented good outcome for most indices. rs-fMRI PCC turned out to have all FN with good outcome, although there were only two. Specifically, comparable rates were found for MD (56%) and FA (50%) as well as T1 (64%) and rs-fMRI PreCun (67%). The lowest good outcome rate was relative to GM (44%) compared with rs-fMRI PCC (100%).

### Visual Interpretation

**Fig. 3:**
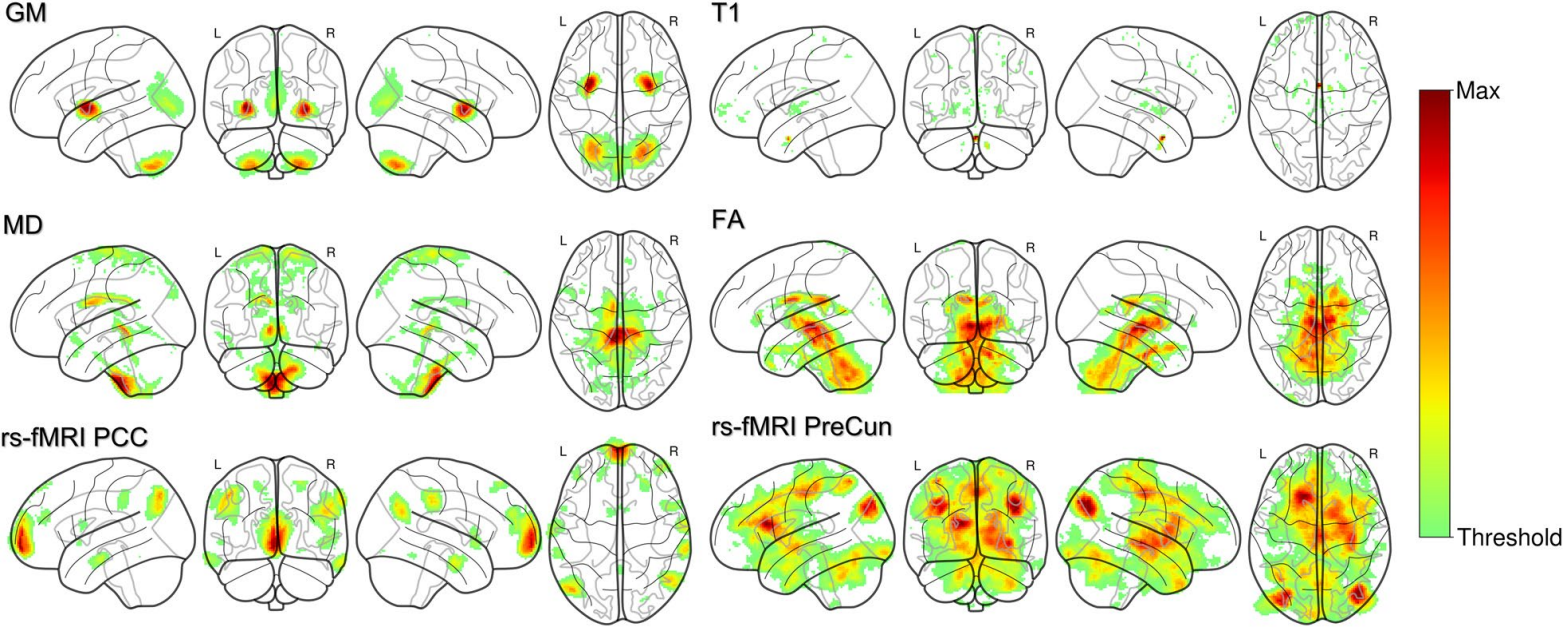
3D CNN visual interpretation. Visualization maps representing activation values from the learned convolutional filters passed over the images. The absolute difference between maps belonging to correctly classified samples of the training set is shown to highlight the most discriminant voxels. To obtain clearer visualizations, we applied a threshold value (Threshold) equal to half of the maximum value (Max) considering activation values from every magnetic resonance (MR) index. Notice how voxels with greater activation vary according to the MR index. FA, fractional anisotropy, GM, gray matter volume, l, left, MD, mean diffusivity, PCC, posterior cingulate cortex, PreCun, precuneus, r, right, rs-fMRI, resting-state functional MRI, T1, T1-weighted

Visualizations maps obtained for each MR index are displayed in Fig. 3. These results were computed considering results on the training set averaged more than tenfold for a single repetition as an emblematic example.

Interestingly, each MR index revealed different parts of the image presenting higher activation values. For example, subcortical brain structures and the brainstem in particular were clearly identified by FA visualizations. It should be noted that functional indices (rs-PCC and rs-PreCun) were related to widespread associative cortical regions, including the mPFC.

## Discussion

Converging evidence suggests that coma is a “disconnection syndrome” [10, 11, 19, 20, 35] because of the combined deleterious effect of primary and secondary severe brain insults. This concept fits well with theoretical frameworks for information processing, according to which higher-order cognitive processing occurs when information is globally available to multiple brain systems, through long-range *functional* interactions, that are intrinsically constrained by brain *structural* connectivity [35, 36]. However, there is an unmet need to find the best way to efficiently analyze structural and functional neuroimaging data to fulfill this knowledge gap and develop highly needed medical decision aid tools for physicians in charge of patients in postanoxic coma. To the extent of our knowledge, we report for the first time successful discrimination of patients in coma from controls using 3D CNN in combination with structural and functional MR indices. Interestingly, the best accuracy was achieved by rs-fMRI PCC amounting to 0.96 (0.94–0.98) on the test set from 10-time repeated tenfold cross-validation. The majority voting strategy also proved how every MR index could contribute, to some extent, to compensate for missing information from the other MR indices, thus obtaining the correct final label.

Developing and validating accurate methods to automatically organize, merge, and analyze raw structural and functional 3D brain MRI data is a mandatory step prior to studies that will specifically focus on the prognostic value of artificial intelligence–empowered neuroimaging data. It is worth noting that as an exploratory goal and an aim to prepare such future neuroprognostication studies, we have specifically reported CNN model errors in relation with patients’ 3-month neurological outcome after CA. Thereby, in line with previous reports [5–9], we observed that resting-state functional connectivity seems to carry critical information that could be used to predict the neurological outcomes of patients in coma in this clinically challenging setting.

Although the success of CNNs for health care use seems promising, one significant limitation is likely to hinder its acceptance by physicians and patients’ next of kin, namely its lack of interpretability. In fact, CNNs are currently described as black boxes that hinder the identification of the most influential features for output classification. Interestingly, we assessed the discriminative value of each MRI indices independently and applied a new voxel-based visualization method built upon the study of the convolutional filters. Overall, structural indices turned out to be less effective in discrimination than functional indices. It is worth noting that the effects of acute severe brain injury on both structural and functional whole-brain connectivity are poorly understood, although the relationship between these connectivity components might not be straightforward [37, 38]. Overall, we think that this result is in line with a previous report from our group, which compared structural and functional MRI data from patients in coma, and which suggested that fMRI data might have a greater contribution for patients’ neuroprognostication than sMRI data [8]. Moreover, it should be noted that, in agreement with current knowledge regarding the functional segregation within the posteromedial parietal cortex [39] and the role of this critical brain hub for conscious processing [40], fMRI data from PCC have shown a greater value in terms of discrimination than PreCun.

Furthermore, a voxel-based study of CNN’s filters contributions allowed us to significantly increase the interpretability of our model. For instance, the FA filter analysis shed light on the well-described relationship between subcortical structure damages and consciousness abolition after CA. In addition, by feeding PPC-centered rs-fMRI input, the visualization maps enabled the discovery that among the most relevant voxels taken into account for CNN discrimination were those situated in the mPFC. This result agrees with previous reports on the potential value of frontoparietal functional disconnections as reliable biomarkers of coma [6–8].

Our results must be interpreted with caution and a few drawbacks should be borne in mind. The first is related to the restricted sample size. This represents one of the major concerns, especially when using deep learning in the medical domain, which is often characterized by either greatly imbalanced classes or lack of sufficient examples. Nevertheless, there exists already some work targeting this issue, demonstrating that deep networks can perform well even with few samples [41]. Another issue regards using 3D CNN architectures instead of more frequently reported 1D or 2D deep learning models. We think that this is an important matter, as the straightforward use of 3D MRI volumes directly as input to the CNN reduces not only preprocessing steps but seems to come with the added value of spatial information. CNN structures with a comparable configuration fed with MRI data have been proven to be very effective in distinguishing various cerebral pathologic conditions, such as neurodegenerative disorders, schizophrenia, and autism [42–46].

A fully automated identification of clinically relevant weak signals from complex multimodal neuroimaging data is a major research topic that may bring a radical paradigm shift for the management of patients in postanoxic coma. In this study, patients in coma were successfully discerned from controls using 3D CNN in combination with different MR indices. The best accuracy was achieved by fMRI data, in particular with rs-fMRI PCC. Furthermore, even more satisfactory performances were achieved through the majority voting strategy, which was able to even out mistakes from single MR indices. A posteriori analysis of misclassified patients indeed indicated that a multimodal MRI approach could be adopted to let the CNN combine information from the totality of MRI indices and even select the most significant ones. This may aid clinicians in establishing prognosis thanks to the potential knowledge-discovery performed by deep learning methods. The proposed approach appears to be feasible and effective, yet is open to further advancements. Future studies are warranted to specifically address the use of these novel approaches for neuroprognostication in patients in coma, probably by developing and validating larger models that could encompass additional clinical standard predictors of neurological recovery.

## Supplementary Information

Below is the link to the electronic supplementary material.

Supplementary file1 (DOCX 131 kb)

Supplementary file2 (DOCX 24 kb)
