# Supplementary material for: Multimodal MRI-Based Whole-Brain Assessment in Patients In Anoxoischemic Coma by Using 3D Convolutional Neural Networks
Source: Neurocrit Care. 2022 Jul 25;37(Suppl 2):303–12. doi: 10.1007/s12028-022-01525-z (PMC9343298; doi:10.1007/s12028-022-01525-z)
Supplement: Supplementary file 1 — Supplementary file1 (DOCX 131 kb) [file 12028_2022_1525_MOESM1_ESM.docx]

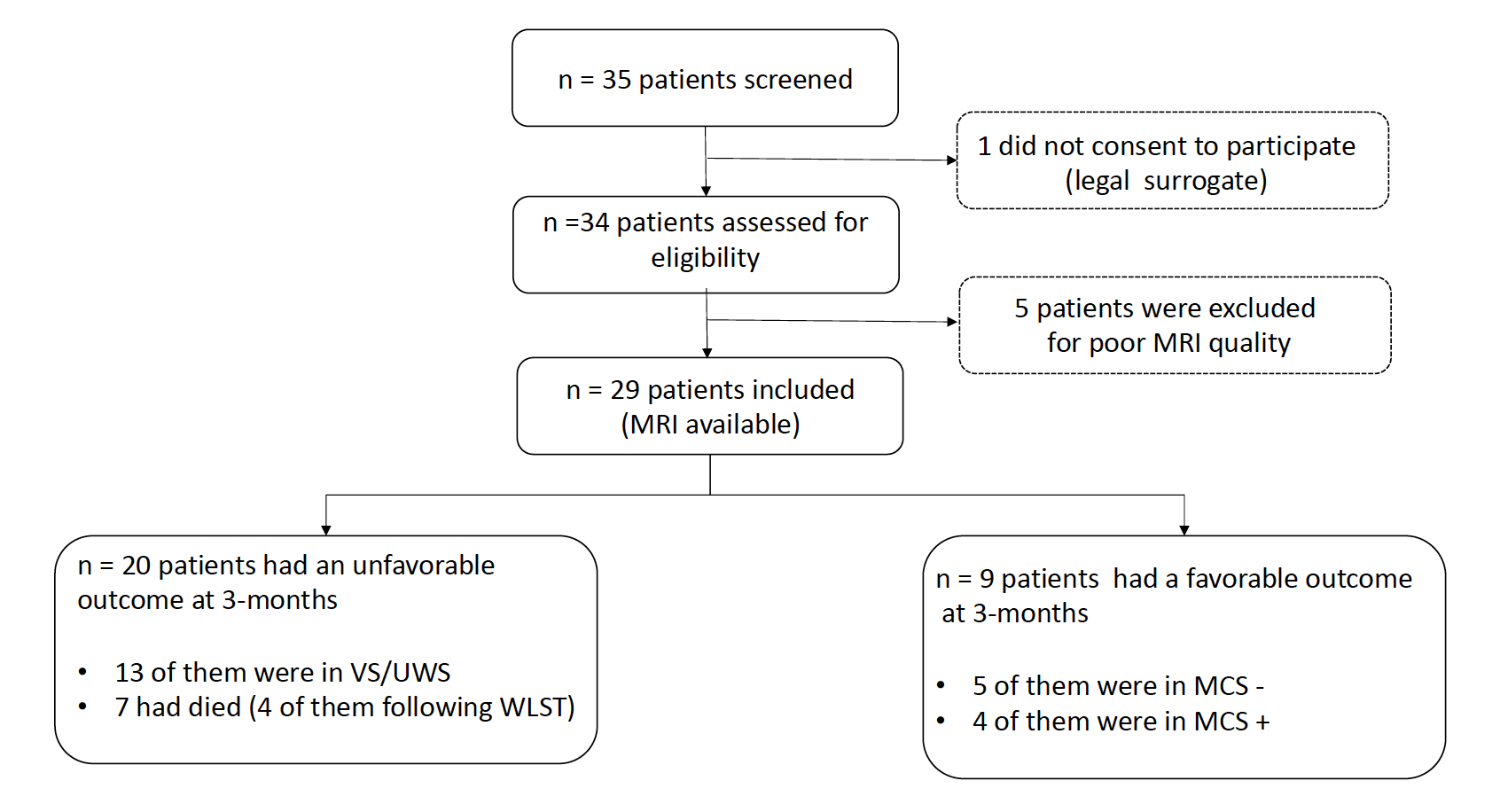


*Supplementary Figure 1.* **Study Flow Chart.** The final cohort consisted of 29 post-anoxic patients and 34 healthy volunteers. Abbreviations: MCS (+) = Minimally Conscious State with command-following, intelligible verbalization or intentional communication; MCS (-) = Minimally Conscious State without command-following, intelligible verbalization or intentional communication; VS/UWS = vegetative state/unresponsive wakefulness syndrome; WLST = withdrawal and limitation of life-sustaining treatments.
