# Supplementary material for: Multimodal MRI-Based Whole-Brain Assessment in Patients In Anoxoischemic Coma by Using 3D Convolutional Neural Networks
Source: Neurocrit Care. 2022 Jul 25;37(Suppl 2):303–12. doi: 10.1007/s12028-022-01525-z (PMC9343298; doi:10.1007/s12028-022-01525-z)
Supplement: Supplementary file 2 — Supplementary file2 (DOCX 24 kb) [file 12028_2022_1525_MOESM2_ESM.docx]

| Patient | Sex | Age, y | Cause | GCS at hospital admission  (subscales) | FOUR at hospital admission | CRS at 3 months  (subscales; DOC state) | Structural MRI findings |
| --- | --- | --- | --- | --- | --- | --- | --- |
| 1 | M | 35 | CA | 6 (1/1/4) | 6 | 9 (2/0/2/2/1/2; VS/UWS) | No abnormality |
| 2 | M | 69 | CA | 4 (1/1/2) | 2 | 4 (1/0/1/1/0/1);  VS/UWS) | Diffuse hypoxic-ischemic injury (thalami, occipital lobes, parietal lobes) |
| 3 | M | 62 | CA | 4 (1/1/2) | 2 | 23  (3/2/3/6/5/4;  MCS+) | Mild increase in signal in thalami and hippocampi |
| 4 | M | 59 | CA | 6 (1/1/4) | 7 | 14  (2/0/2/4/3/3);  MCS-) | No abnormality |
| 5 | F | 46 | CA | 5 (1/1/3) | 5 | 21  (3/2/3/5/4/4;  MCS+) | No abnormality |
| 6 | F | 49 | CA | 3 (1/1/1/) | 1 | 6  (2/0/1/1/1/1;  VS/UWS) | Diffuse ischemic changes (thalami, occipital lobes) |
| 7 | F | 56 | CA | 5 (1/1/3) | 6 | 8  (2/0/1/2/1/2;  VS/UWS) | No abnormality |
| 8 | F | 61 | CA | 5 (1/1/3) | 4 | 13  (2/0/1/4/3/3;  MCS+) | High signal in thalami bilaterally |
| 9 | M | 58 | CA | 6 (1/1/4) | 5 | 12  (2/0/2/3/3/2;  MCS-) | No abnormality |
| 10 | F | 22 | CA | 6 (1/1/4) | 6 | 7 (2/0/1/2/1/1; VS/UWS) | No abnormality |
| 11 | F | 53 | CA | 5 (1/1/3) | 4 | NA (died) | High signal of the thalami bilaterally |
| 12 | M | 56 | CA | 6 (1/1/4) | 6 | NA (died) | No abnormality |
| 13 | M | 49 | CA | 5 (1/1/3) | 4 | 7  (2/0/1/2/1/1;  VS/UWS) | High increase in signal in thalami and hippocampi |
| 14 | M | 78 | CA | 6 (1/1/4) | 7 | 7  (2/0/1/1/1/2;  VS/UWS) | Mild increase in signal in thalami and hippocampi |
| 15 | M | 65 | CA | 6 (1/1/4) | 7 | 16  (3/2/3/3/2/3;  MCS+) | High signal of the thalami bilaterally |
| 16 | F | 57 | CA | 4 (1/1/2) | 2 | 8  (2/0/2/2/1/1;  VS/UWS) | No abnormality |
| 17 | M | 49 | CA | 5 (1/1/3) | 5 | NA (died) | Diffuse ischemic changes (thalami, occipital lobes) |
| 18 | F | 55 | CA | 4 (1/1/2) | 2 | 8  (2/0/2/2/1/1;  VS/UWS) | Mild increase in signal in thalami and hippocampi |
| 19 | F | 61 | CA | 5 (1/1/3) | 6 | 12  (2/0/2/3/3/2);  MCS-) | High signal of the thalami bilaterally |
| 20 | M | 62 | CA | 5 (1/1/3) | 5 | 12 (2/0/2/3/3/2;  MCS-) | No abnormality |
| 21 | M | 58 | CA | 5 (1/1/3) | 5 | NA (died) | Diffuse hypoxic-ischemic injury (thalami, occipital lobes, parietal lobes) |
| 22 | M | 48 | CA | 5 (1/1/3) | 6 | 8 (2/0/1/2/1/2;  VS/UWS) | Diffuse ischemic changes (thalami, occipital lobes) |
| 23 | M | 52 | CA | 4 (1/1/2) | 5 | NA (died) | No abnormality |
| 24 | F | 65 | CA | 5 (1/1/3) | 6 | 8 (2/0/1/2/1/2;  VS/UWS) | High signal of the thalami bilaterally |
| 25 | M | 60 | CA | 4 (1/1/2) | 2 | 7 (2/0/1/2/1/1;  VS/UWS) | No abnormality |
| 26 | M | 76 | CA | 5 (1/1/3) | 5 | 7 (2/0/1/2/1/1;  VS/UWS) | Diffuse hypoxic-ischemic injury (thalami, occipital lobes, parietal lobes) |
| 27 | M | 42 | CA | 3 (1/1/1) | 1 | NA (died) | No abnormality |
| 28 | M | 43 | CA | 4 (1/1/2) | 2 | NA (died) | High increase in signal in thalami and hippocampi |
| 29 | F | 38 | CA | 5 (1/1/3) | 4 | 13 (3/0/2/3/3/2;  MCS-) | High signal in thalami bilaterally |

*Supplementary Table 1.* **Demographic data**. Abbreviations: GCS = Glasgow Coma Scale; FOUR = Full Outline of UnResponsiveness; CRS-R = Coma Recovery Scale Revised; VS/UWS = Vegetative State/Unresponsive Wakefulness Syndrome; MCS (+) = Minimally Conscious State with command-following, intelligible verbalization or intentional communication; MCS (-) = Minimally Conscious State without command-following, intelligible verbalization or intentional communication; CA = Cardiac Arrest; DOC = disorder of consciousness. GCS subscales = eyes opening / verbal response / motor response. CRS-R subscales = arousal / communication scale / oromotor and verbal functions / motor functions / visual functions / auditory functions.
